# Supplementary material for: Causal Relationship between Aging and Anorexia Nervosa: A White-Matter-Microstructure-Mediated Mendelian Randomization Analysis
Source: Biomedicines. 2024 Aug 16;12(8):1874. doi: 10.3390/biomedicines12081874 (PMC11351342; doi:10.3390/biomedicines12081874)
Supplement: Supplementary file 1 [file biomedicines-12-01874-s001.zip › Supplementary Figure/Figure S4/Singe SNP plot of MR analysis for TL to ubm-b-1969.pdf]

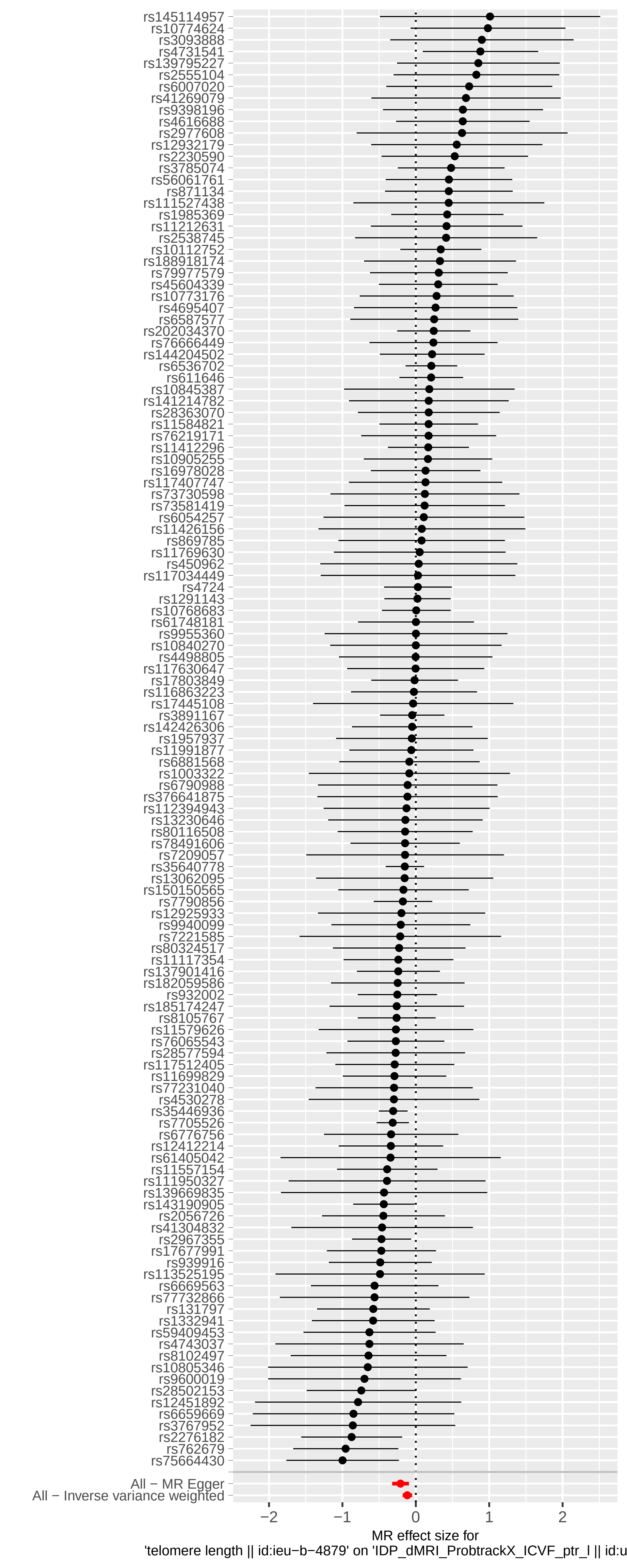
rs145114957
rs10774624
rs3093888
rs4731541
rs139795227
rs2555104
rs6007020
rs41269079
rs9398196
rs4616688
rs2977608
rs12932179
rs2230590
rs3785074
rs56061761
rs871134
rs111527438
rs1985369
rs11212631
rs2538745
rs10112752
rs188918174
rs79977579
rs45604339
rs10773176
rs4695407
rs6587577
rs202034370
rs76666449
rs144204502
rs6536702
rs611646
rs10845387
rs141214782
rs28363070
rs11584821
rs76219171
rs11412296
rs10905255
rs16978028
rs117407747
rs73730598
rs73581419
rs6054257
rs11426156
rs869785
rs11769630
rs450962
rs117034449
rs4724
rs1291143
rs10768683
rs61748181
rs9955360
rs10840270
rs4498805
rs117630647
rs17803849
rs116863223
rs17445108
rs3891167
rs142426306
rs1957937
rs11991877
rs6881568
rs1003322
rs6790988
rs376641875
rs112394943
rs13230646
rs80116508
rs78491606
rs7209057
rs35640778
rs13062095
rs150150565
rs7790856
rs12925933
rs9940099
rs7221585
rs80324517
rs11117354
rs137901416
rs182059586
rs932002
rs185174247
rs8105767
rs11579626
rs76065543
rs28577594
rs117512405
rs11699829
rs77231040
rs4530278
rs35446936
rs7705526
rs6776756
rs12412214
rs61405042
rs11557154
rs111950327
rs139669835
rs143190905
rs2056726
rs41304832
rs2967355
rs17677991
rs939916
rs113525195
rs6669563
rs77732866
rs131797
rs1332941
rs59409453
rs4743037
rs8102497
rs10805346
rs9600019
rs28502153
rs12451892
rs6659669
rs3767952
rs2276182
rs762679
rs75664430
All − MR Egger
All − Inverse variance weighted
−2
−1
0
1
2
MR effect size for
'telomere length || id:ieu−b−4879' on 'IDP_dMRI_ProbtrackX_ICVF_ptr_l || id:u
